# Supplementary material for: Structural basis for guide RNA selection by the RESC1–RESC2 complex
Source: Nucleic Acids Res. 2023 Mar 31;51(9):4602–12. doi: 10.1093/nar/gkad217 (PMC10201420; doi:10.1093/nar/gkad217)
Supplement: gkad217_Supplemental_File [file gkad217_supplemental_file.pdf]

Supplementary Information

Structural basis for guide RNA selection by the RESC1-RESC2 complex

Luciano G. Dolce[1], Yevheniia Nesterenko[1], Leon Walther[1], Félix Weis[2,⊥], and Eva Kowalinski[1,*]

[1] EMBL Grenoble, 71 Avenue des Martyrs, 38042 Grenoble, France

[2] EMBL Heidelberg, Structural and Computational Biology Unit, Meyerhofstraße 1, 69117 Heidelberg, Germany

[⊥] Current address: Institut de Biologie Structurale, 71 Avenue des Martyrs, 38000 Grenoble, France

[*]Corresponding author email: kowalinski@embl.fr

Key words: RESC; RNA editing; cryo-EM structure; *Trypanosoma brucei*

Supplementary figure S1: Biochemical and structural characterisation of RESC1-RESC2 heterodimer. **(A)** Refeyn mass photometry measurement of RESC1-RESC2 molecular weight in solution, indicating an approximately 100 kDa particle. **(B)** Example of size exclusion chromatography used as a final step of the purification of RESC1-RESC2, from a S200 16/600 GL column. **(C)** Cartoon representation of the Alphafold prediction of RESC1-RESC2, coloured by pLDDT using the alphafold palette, with corresponding PAE plot. **(D)** Alphafold prediction fitted in the deepEMhancer post-process EM map by ChimeraX, with no density for the flexibly attached N-terminal domains. **(E and F)** Detail of the dimerisation interfaces β-harpin in RESC1 and RESC2, with the deepEMhancer post-process EM map around the β-harpin represented as blue mesh. The lower quality of the EM map in the region of RESC1's hairpin suggests the loop to be more flexible than in RESC2. **(G, H and I)** Example of the deepEMhancer post-process EM map, as a blue mesh, of a helix and two strands, with sticks coloured by heteroatom. **(J)** Dimerisation interface in RESC1, with RESC1 represented as surface and coloured by coulombic electrostatic potential (blue positive and red negative) calculated by ChimeraX, and RESC2 as cartoon. **(K)** Dimerisation interface in RESC2, with RESC2 represented as surface and coloured by coulombic electrostatic potential (blue positive and red negative) calculated by ChimeraX, and RESC1 as cartoon. **(L)** 4 views of the RESC1-RESC2 model fit in the final deepEMhancer post-process EM map.

Supplementary figure S2: Processing scheme of the cryo-EM structure determination of RESC1-RESC2. **(A)** Example of a representative micrograph, resulting from the averaging of the motion-corrected frames of a movie. **(B)** Example of 2D classes made with particles picked by warp. **(C)** Particle sorting process by ab-initio and heterogeneous refinements. **(D)** Polishing steps of the final particle stack, followed by post-processing in Relion or with DeepEMhancer. **(E)** Local resolution map, as calculated by Relion and displayed in ChimeraX. **(F)** Angular distribution map of particle orientation of the final refinement step in Relion. Map recalculated in cryoSPARC for representation proposes. **(G)** Final resolution of the reconstruction, as calculated by Relion Gold Standard Fourier Shell Correlation, with a resolution of 3.4 Å when adopting a cutoff of 0.143.

Supplementary figure S3: Sequence alignment of RESC1 and RESC2 from different kinetoplastida members. Start residue of the RESC1 and RESC2 constructs annotated as yellow diamonds, start residue of cryo-EM based model annotated by green and blue triangle for RESC1 and RESC2 respectively. Flexible attached N-terminal domains are indicated in pink. Dimerization β-harpin coloured as green and blue, for RESC1 and RESC2, respectively. Signature motifs (a, b and c) of the divalent cation-dependent family of RNA triphosphatases are indicated. The four observed 5'- triphosphate binding residues in RESC2 are indicated with a red star, the RESC2$^{K311S/R402D}$ mutant residues as a red star inside a circle. Red boxes indicate 100% conservation of RESC1 and RESC2 in all shown species.

Supplementary figure S4: Biochemical characterisation of RESC1-RESC2 5' triphosphate binding. **(A)** Analytical size exclusion chromatography of RESC1-RESC2 with gRNA, with or without 5' triphosphate. Control experiments for Figure 2G are included. Chromatograms, full images of the SDS and Urea PAGEs. **(B)** Size exclusion titration of different molar ratios of gRNA on RESC1-RESC2. No further peak shift is detected above 1:1 gRNA:protein ratio. **(C)** SEC-MALS of the RESC1-RESC2 complex with gRNA in a 1:4 gRNA:protein ratio. The calculated

molar mass of the particles were 18.6 ± 0.2 kDa, 102 ± 1 kDa and 113 ± 1 kDa for the gRNA, RESC1-RESC2 and their complex, respectively.

Supplementary figure S5: Processing scheme of the cryo-EM structure determination of RESC1-RESC2 bound to 5' triphosphate gRNA. (A) Example of a representative micrograph, resulting from the averaging of the motion-corrected frames of a movie. **(B)** Example of 2D classes, particles picked by warp. **(C)** Particle sorting process by ab-initio and heterogeneous refinement. **(D)** Polishing steps of the final particle stack, followed by post-processing, in Relion or with DeepEMhancer. **(E)** The local resolution map, as calculated by Relion and displayed in ChimeraX, indicates that the triphosphate-bound tunnel has one of the highest resolutions of the reconstruction. **(F)** Angular distribution map of particle orientation of the final refinement step done in Relion. Map recalculated in cryoSPARC for representation proposes. **(G)** Final resolution of the reconstruction, as calculated by Relion Gold Standard Fourier Shell Correlation, with a resolution of 4.7 A when adopting a cutoff of 0.143.

Supplementary figure S6: Difference between bound and apo maps of RESC1-RESC2. To cross-validate our approach of generating a subtraction map in Chimera, we generate a difference map (bound minus apo) using the TEMPy Difference Map program implemented in CCP-EM, which uses an automated multistep protocol to produces relatively clean and interpretable difference maps. The program was run using the default parameters. (Joseph et al. J. Chem. Inf. Model. 2020, 60, 5, 2552–2560 https://doi.org/10.1021/acs.jcim.9b01103) **(A)** Cartoon model of RESC1-RESC2 superimposed with the TEMPy Difference Map between bound and unbound RESC1-RESC2 (in grey), with the GTP shown in sticks and coloured by heteroatom, indicating additional density exclusively inside the triphosphatase tunnel of RESC2. **(B and C)** Two orthogonal views of the triphosphatase tunnel of RESC2, with a GTP fitted in the TEMPy Difference Map (blue mesh). Lysine and arginine side chains, that coordinate the γ-phosphate of the GTP, are shown in sticks and coloured by heteroatom.

Supplementary figure S7: Biochemical characterisation of RESC2 5' triphosphate binding. (A) Analytical size exclusion chromatograms of RESC1^{wt}-RESC2$^{K311S/R402D}$ mutant complex, complementing the Figure 3D, with the controls, and the full image of the SDS and Urea PAGE. **(B)** Analytical size exclusion chromatograms of RESC1^{wt}-RESC2^{K311S} complex. gRNA binding in this single mutant was affected but not abolished. This experiment and related controls were performed in a buffer containing 400 mM NaCl.

Supplementary Figure S1

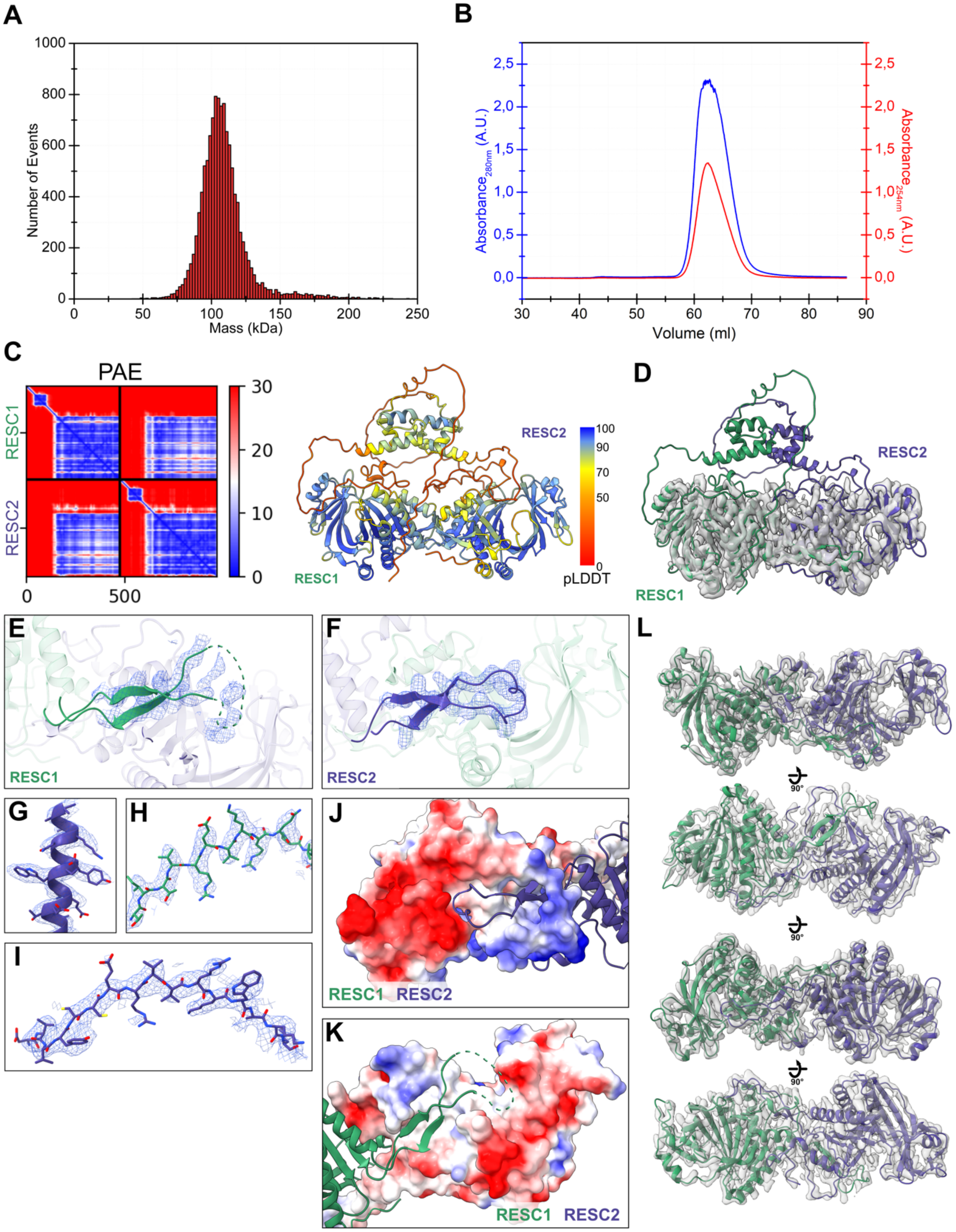

Supplementary Figure S2

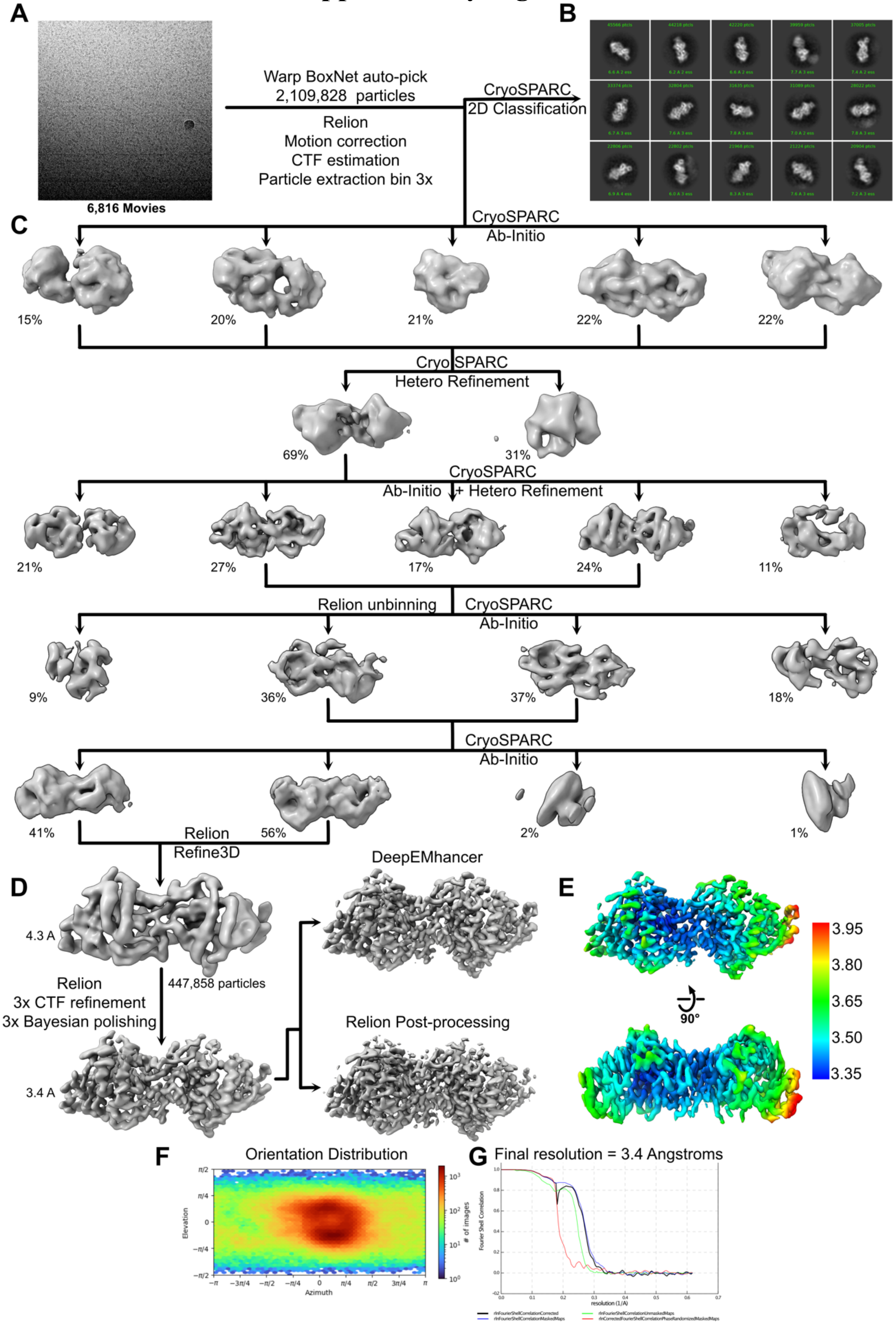

Supplementary Figure S3

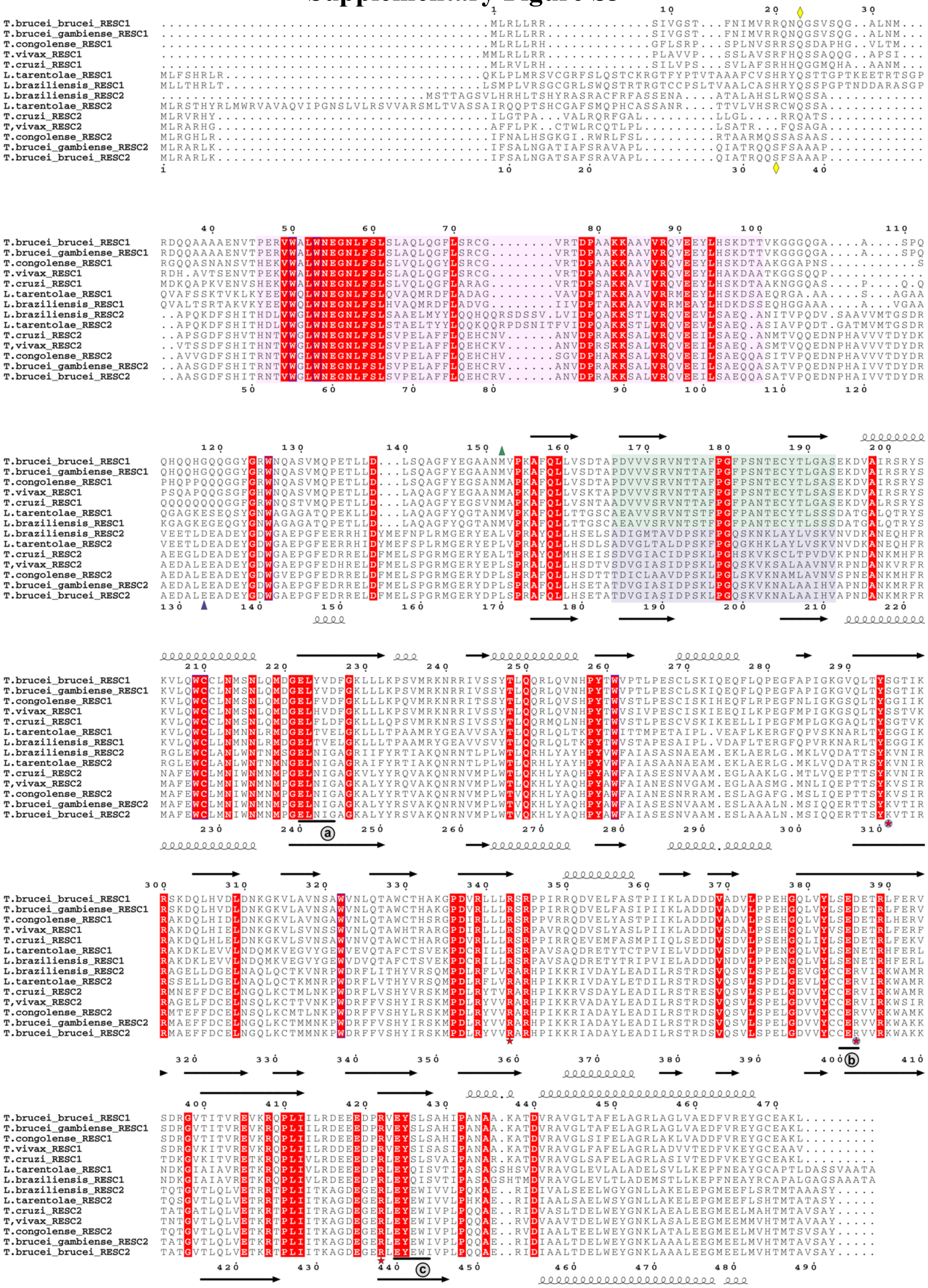

Supplementary Figure S4

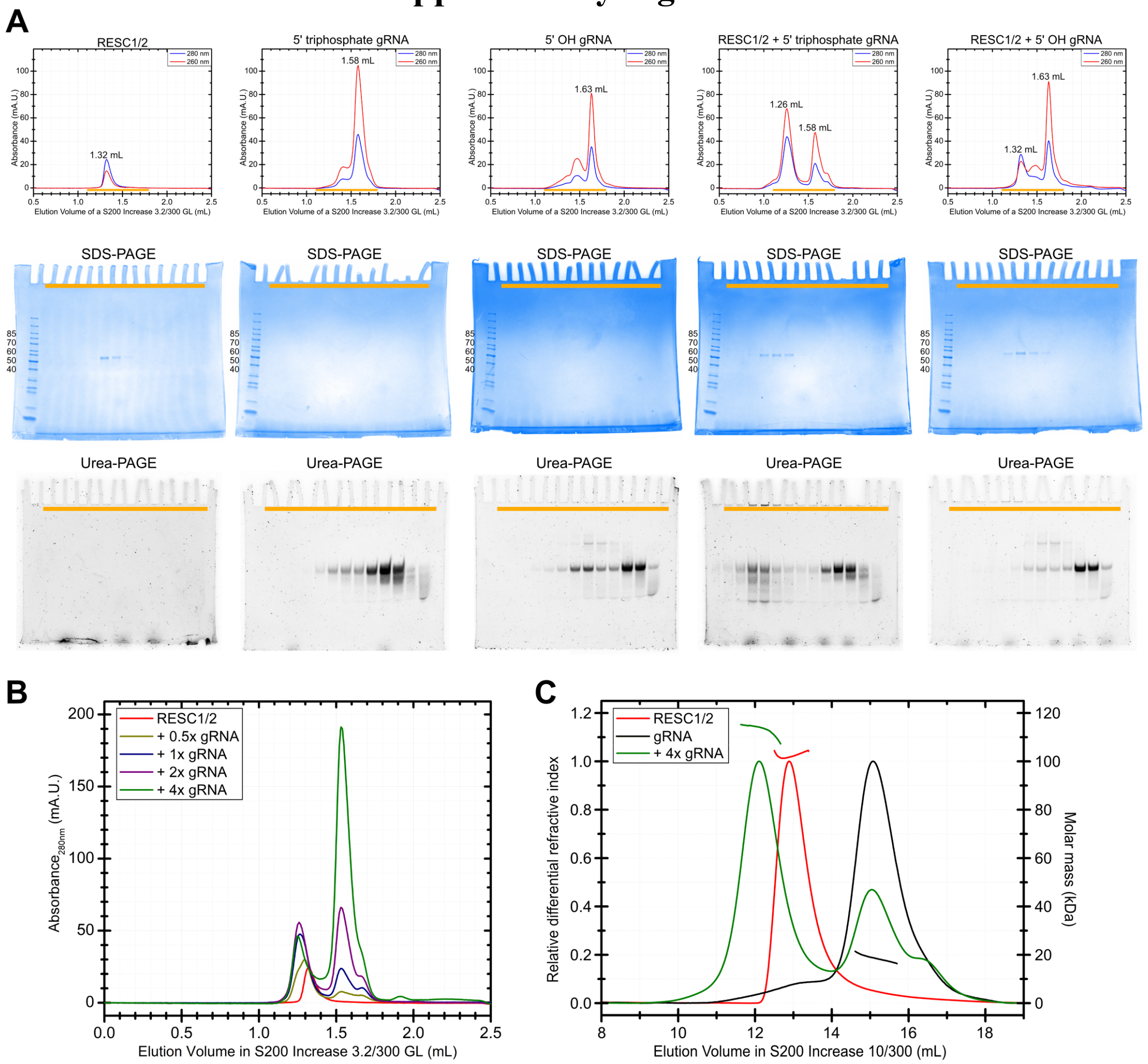

Supplementary Figure S5

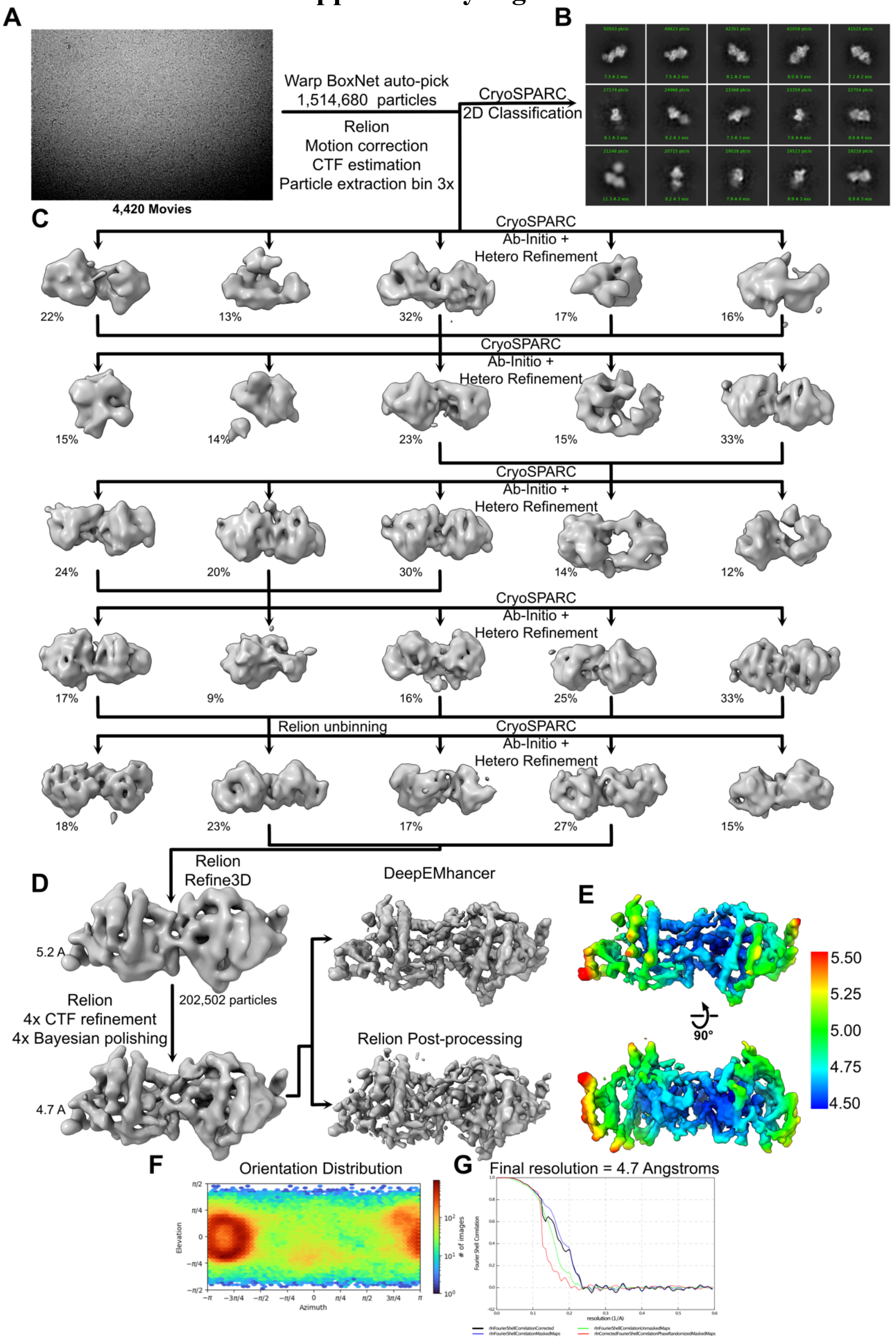

Supplementary Figure S6

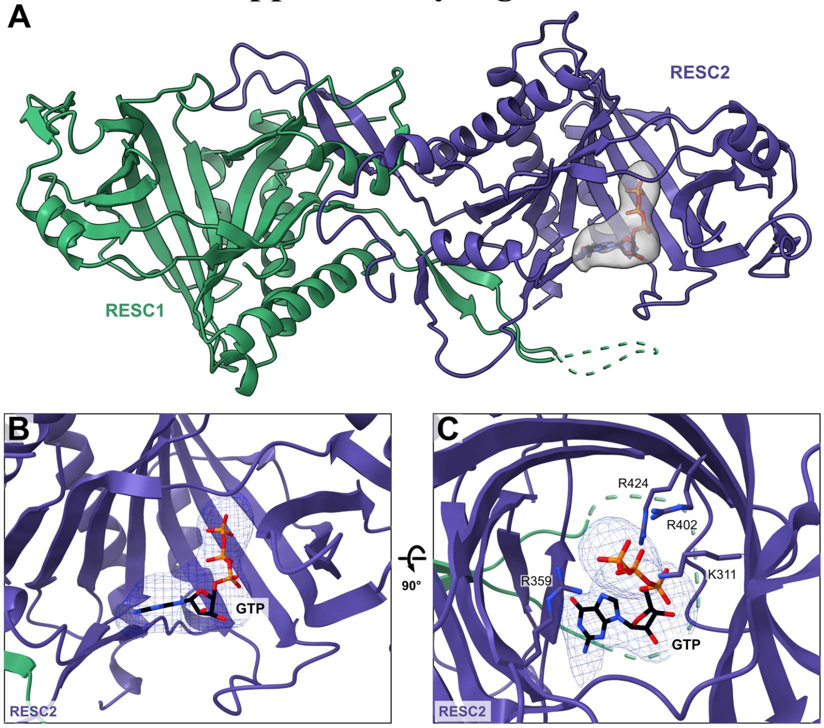

Supplementary Figure S7

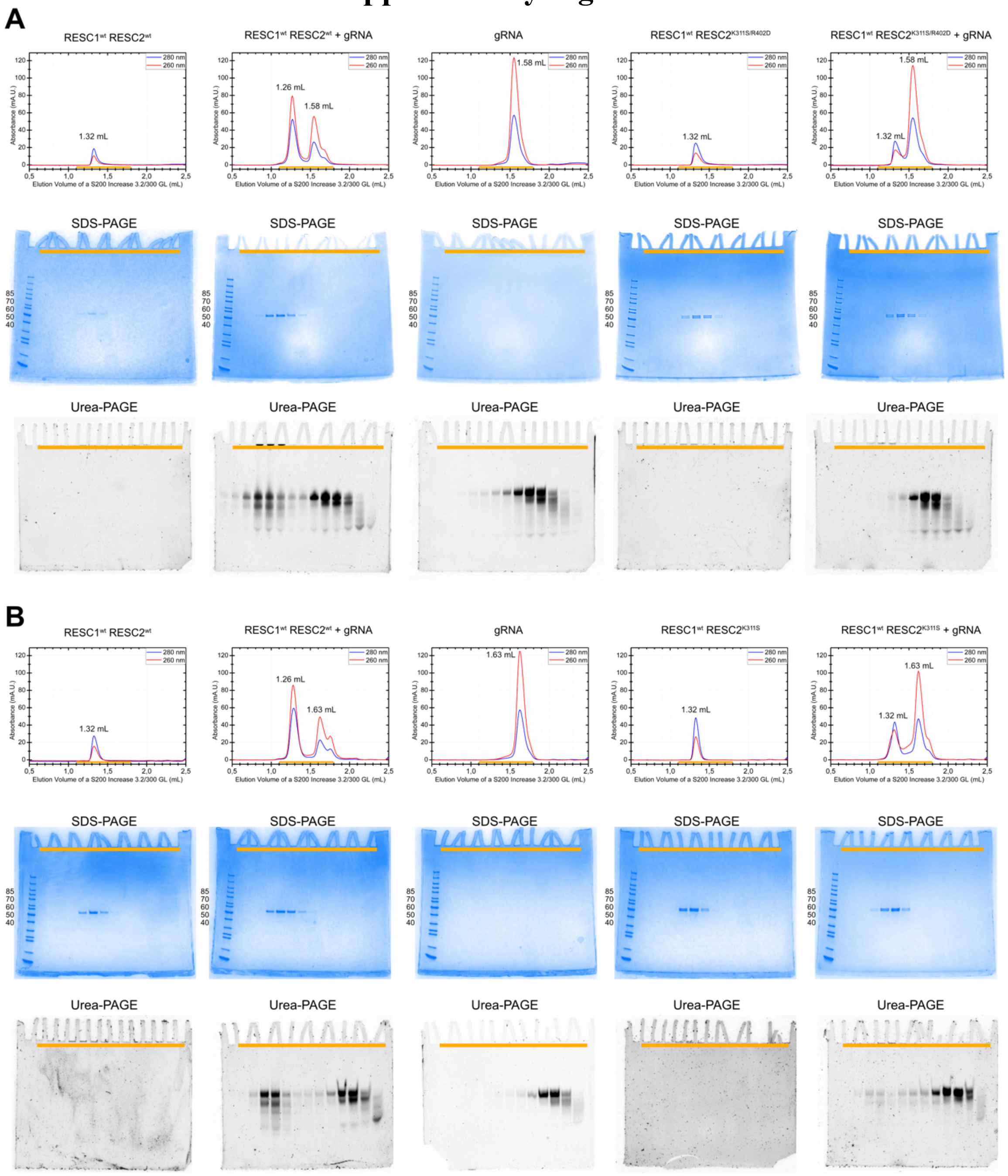

Supplementary Table 1. Summary of Cryo-electron microscopy data collection and refinement statistics

**Data Collection**	**RESC1-RESC2**	**RESC1-RESC2 + gRNA**
Microscope	Titan Krios	Titan Krios
Voltage	300 kV	300 kV
Camera	Quantum-K2	K3
Energy filter	GIF	Quantum-LS
Spot size	8	8
Beam size	650 nm	1100 nm
Pixel size (A/pix)	0.81	0.84
Preset defocus range	-1.2 to -2.5	-1.2 to -3.5
Stage tilt	30 deg	30 deg
Dose rate (e-/pixel.s) / total exposure (e-/A2)	3 / 63	19.3 / 62.9
Number of frames per movie	100	60
Number of movies	6,145	4,420
**Data Processing**	**RESC1-RESC2**	**RESC1-RESC2 + gRNA**
Initially warp autopick particles	2,109,828	1,514,680
Final number of particles	447,858	202,502
Resolution FSC	3.4	4.7
Sharpening method	DeepEMhancer	DeepEMhancer
EMDB accession number	EMD-16592	EMD-16593
**Refinement**	**RESC1-RESC2**	
PDB accession number	8CDP	
No atoms	5200	
Residues (protein)	654	
CCbox, CCmask, CCvolume	0.89, 0.83, 0.83	
R.M.S.D.		
Bond lengths	0.003	
Bond angles	0.620	
Ramachandran favoured (%)	97.52	
Ramachandran allowed (%)	2.48	
Ramachandran outlier (%)	0	
MolProbity score	1.69	
Clash score	12.00	
